# Static Plantar Pressure Distribution Measured Using PodoPrint Aluminum Platform in Multiple Sclerosis Patients: A Case–Control Study

**DOI:** 10.1002/jfa2.70061

**Published:** 2025-07-01

**Authors:** Francisco Javier Ruiz‐Sánchez, Maria do Rosário Martins, Marta Elena Losa‐Iglesias, Ricardo Becerro‐de‐Bengoa‐Vallejo, Juan Gómez‐Salgado, Miguel Ángel Saavedra‐García, Daniel López‐López, Ana María Jiménez‐Cebrián

**Affiliations:** ^1^ Department of Health Sciences Faculty of Nursing and Podiatry Industrial Campus of Ferrol Research Health and Podiatry Group Universidade da Coruña Ferrol Spain; ^2^ UICISA:E; Instituto Politécnico de Viana do Castelo Viana do Castelo Portugal; ^3^ Faculty of Health Sciences Universidad Rey Juan Carlos Madrid Spain; ^4^ Faculty of Nursing Physiotherapy and Podiatry Universidad Complutense de Madrid Madrid Spain; ^5^ Department of Sociology Social Work and Public Health Universidad de Huelva Huelva Spain; ^6^ Safety and Health Postgraduate Programme Universidad Espíritu Santo Guayaquil Ecuador; ^7^ Grupo de Investigación en Ciencias del Deporte (INCIDE) Universidade da Coruña A Coruña Spain; ^8^ Nursing and Podiatry Department Faculty of Health Sciences Instituto de Investigación Biomédica de Málaga (IBIMA) University of Malaga Malaga Spain

**Keywords:** foot, lower limb, multiple sclerosis, plantar force

## Abstract

**Introduction:**

Multiple sclerosis (MS) is a complex early‐onset neurological disorder with significant functional implications. Neuroinflammation and neurodegeneration are the primary pathological processes underlying MS, both of which may alter lower limb loading patterns. These alterations can be evaluated through static plantar pressure distribution. This study hypothesized that patients with MS would exhibit higher static plantar pressures compared to healthy controls.

**Methods:**

A multicenter case‐control study was conducted with 88 participants, comprising 44 patients diagnosed with MS and 44 age‐ and sex‐matched healthy controls. Static plantar pressures were assessed using the PodoPrint Aluminum pressure platform.

**Results:**

The MS group demonstrated significantly lower maximum peak pressure values in both the right foot (*p* < 0.007) and the left foot (*p* < 0.001) compared to the control group.

**Conclusion:**

A marked reduction in maximum peak pressures and weight‐bearing capacity was observed in patients with MS may have reduced plantar pressure, which could be indicative of altered gait patterns and balance issues.

## 1 Introduction

Multiple sclerosis (MS) is a complex disease that affects patients in the early years of adulthood and significantly impacts their quality of life [1]. Walking difficulties are common in patients with MS, and several factors, such as ataxia, hypertonic muscles, and secondary musculoskeletal deformities, interfere with normal plantigrade foot contact during walking [2].

Understanding the gait cycle is crucial for differentiating possible deficiencies in patients with MS. The gait cycle consists of the stance phase (approximately 60%) and the swing phase (approximately 40%). The stance phase is further divided into several stages: loading response, midstance, and heel off, whereas the swing phase includes initial and terminal swing. There are two brief moments of double support when both feet touch the ground, allowing for weight transfer from the rear foot to the front foot. A single limb supports body weight for approximately 80% of the gait cycle [3].

Neuroinflammation and neurodegeneration are the two processes that physiopathologically define MS. Although movement disorders are common in MS, their precise pathophysiological basis remains elusive [4]. The most common movement disorders in MS are restless legs syndrome, tremors, ataxia, parkinsonism, paroxysmal dyskinesias, and dystonia, all of which can significantly impact patients' quality of life [5]. Parkinsonism in MS is a rare but recognized manifestation that may arise due to the involvement of dopaminergic pathways and the basal ganglia [1]. This secondary manifestation is often difficult to distinguish from primary parkinsonism such as Parkinson's disease [2]. Despite its infrequency, parkinsonism can be relevant for some patients with MS. Ataxia is a common manifestation in demyelinating disorders, affecting nearly 80% of patients with MS, and has a significant impact on health‐related quality of life (HRQoL) [6]. Evidence suggests that patients with ataxia exhibit greater gait variability compared to healthy controls [7], leading to a negative impact on their quality of life. This is supported by findings indicating that patients with MS have a lower quality of life compared to healthy subjects due to foot‐related problems [8].

Gait requires a complex biomechanical assessment to identify specific patterns in multiple sclerosis (MS) and to develop effective prophylactic and rehabilitation programs for gait disorders in these patients. Furthermore, MS has a significant effect on gait [9]. Gait impairment in patients with MS is associated with spatiotemporal parameters such as slower walking speed, decreased cadence, shorter stride length, longer stride duration, and increased double support time [9]. These deficiencies lead to reduced postural control, resulting in instability and a variety of limitations for patients with MS [10]. This reduced postural control contributes to gait limitations, such as decreased ankle dorsiflexion [11].

Plantar pressure distribution is a crucial method for biomechanical evaluation. The distribution and magnitude of plantar pressures provide useful and objective information about the effect of hypertonia on foot mechanics and disease progression [12]. Hypertonia, a prevalent chronic symptom in MS, significantly affects the quality of life and functionality of patients. Epidemiological studies have indicated that it can affect up to 80% of patients with MS. Hypertonia manifests as increased muscle stiffness, often accompanied by spasms and altered reflexes [13]. Additionally, it affects normal plantigrade foot contact by altering its accommodation to the ground [14]. However, the impact of hypertonia and foot drop on plantar pressure distribution and balance remains unclear.

The foot, which bears the body's weight and plays a crucial role in locomotion, provides a foundation for balance [15]. Balance is maintained through sensory inputs, particularly proprioception, which is derived from receptors in the joints, muscles, tendons, and ligaments. Proprioception constitutes the perception of the position of the limbs and body [16]. Loss of sensation on the plantar surface of the feet has been associated with decreased balance and an increased risk of falls [17]. In MS, as mentioned earlier, the support base changes, hindering walking and the performance of daily activities [18]. These changes in the support base alter the distribution of plantar load, with excess load on the forefoot associated with balance disorders and falls [19].

Studying plantar pressures in people with multiple sclerosis (MS) is crucial for understanding their pathology in static posture and extrapolating findings to dynamic conditions. This knowledge helps determine when and how to intervene with treatments aimed at improving quality of life. Currently, there is limited literature on how MS affects static plantar pressures and how these compare to healthy patients of the same age, sex, BMI, and geographical location. In this study, static plantar pressures will be compared between patients with MS and healthy subjects, considering variables, such as maximum pressure, contact area, and body weight, supported on each foot. Additionally, the association with common MS pathologies, such as foot drop and hypertonia, will be evaluated.

The introduction provides a detailed and up‐to‐date review of the literature on gait disorders in patients with multiple sclerosis (MS), highlighting the pathophysiological factors that affect motor control and balance, such as neuroinflammation and neurodegeneration. The main clinical manifestations of MS that impact gait, such as ataxia, hypertonia, and movement disorders, are discussed. Additionally, the relevance of plantar pressure distribution as a key biomechanical evaluation tool for understanding changes in foot support patterns is addressed.

The novelty of this study lies in its analysis of static plantar pressure distribution, an aspect that has been underexplored in patients with MS, as most previous studies have focused on dynamic gait. This static approach allows for a more accurate assessment of load distribution under nondynamic conditions. Additionally, the PodoPrint Aluminum platform is used for the first time, offering high sensitivity in measuring plantar pressure distribution, which contributes to a more detailed evaluation of biomechanical alterations in patients with MS. This study also explores the relationship between changes in plantar pressure and common clinical conditions in MS, such as hypertonia and foot drop, providing a more integrated view of the relationship between biomechanical findings and the clinical manifestations of the disease.

Our study hypothesizes that patients with MS exhibit higher plantar pressures than healthy controls. This study explores the relationship between MS and static plantar pressures, considering various factors such as disease characteristics, movement disorders, hypertonia, balance impairment, and demographic variables.

## 2 Materials and Methods

### 2.1 Design and Sample

The sample size calculation was performed to detect differences between two independent groups using the G*Power 3.1.9.2 software. The Mann–Whitney U test was applied with a normal parent distribution, a two‐tailed hypothesis, a large effect size of 0.80, an *α* error probability of 0.05, a *β* level of 20%, and a desired power of 80% (1‐*β* error probability), with an allocation ratio (N2/N1) of 1 [20]. A total sample size of 54 participants was calculated, with at least 27 participants per group. Finally, 88 participants were included in the study: 44 with MS and 44 healthy matched controls.

Volunteers for this study were recruited using a consecutive sampling method, specifically a successive and nonrandomized approach. The study participants included patients with MS who attended their scheduled appointments with their specialist doctors. Controls were matched based on age, sex, foot size, and BMI and were assessed using the same instruments and protocol as the MS group. These controls were recruited from a podiatric medical center in the same town, which specializes in foot care. The study was conducted from October 2023 to January 2024 and adhered to the guidelines outlined in the Helsinki Declaration. It was approved by the Ethics Committee of Provincial Research of Malaga under registration number CEUMA: 32‐2021‐H. All participants provided informed consent for their inclusion in the research. All subjects were assessed using the Expanded Disability Status Scale (EDSS) [21], a tool developed by neuroscientist John F. Kurtzke to evaluate the degree of disability in patients with MS. The EDSS assigns a numerical score ranging from 0 (no disability) to 10 (death from MS), with higher scores indicating greater levels of disability. The score is determined by assessing neurological function in specific areas, such as walking ability, coordination, visual function, and other functions related to the nervous system. The EDSS assists physicians in evaluating disease progression, guiding treatment decisions, and providing a standardized measure of disability in patients with MS [22, 23]. Exclusion criteria included severe mental illness, a high degree of disability, difficulty walking, and the need for devices to maintain bipedal posture [24, 25, 26].

### 2.2 Study Protocol and Data Collection

The data were collected using a pressure platform, which presented challenges due to the gait alterations experienced by patients with MS, making it difficult for them to remain still during the assessment. To ensure accurate data acquisition, a specific protocol was followed:

1. Positioning: The subject was instructed to stand on the pressure platform, positioning their feet at the normal angle of gait. Clear instructions were provided to help the patient achieve the correct stance.
2. Stabilization: Once positioned, the patient was asked to remain still for six seconds, with relaxed shoulders and arms, and to focus on looking straight ahead. This stabilization period was crucial as it allowed the platform to accurately collect plantar pressure data.
3. Data Collection: Three measurements were taken consecutively, and the results were recorded by the lead podiatrist researcher. This approach ensured consistency and reliability in the data collected.
4. Repetition: If the patient altered their initial position during the measurement, the data were deemed invalid and the test was repeated. This step was essential to maintain the accuracy and validity of the measurements. Additionally, precautions were taken to minimize subject fatigue, including providing adequate rest periods between measurements (ranging from 2 to 5 min), ensuring a comfortable testing environment, and monitoring subjects for signs of fatigue throughout the testing process.

This protocol was designed to mitigate the difficulties associated with MS‐related gait alterations and to ensure the collection of reliable plantar pressure data.

Additionally, a thorough physical examination of the feet was performed, including structural assessment through palpation, evaluation of joint mobility, and muscle strength testing. Furthermore, each patient's clinical history was reviewed to identify any other foot pathologies or chronic diseases, and complementary tests, such as ultrasound and X‐rays, were conducted by the lead podiatrist researcher. These alterations included hallux valgus, claw toes, metatarsalgia, heel pain, plantar fasciitis, Morton's neuroma, cavus foot, flat foot, foot drop, equinus deformity, corns, and calluses. The assessment primarily focused on evaluating ankle dorsiflexion. Ankle dorsiflexion was measured with the knee extended and then with the knee flexed using a goniometer. One arm of the goniometer was placed on the lateral and plantar aspects of the heel, whereas the other arm was aligned with the bisector of the tibia, ensuring the subtalar joint was in a neutral position and avoiding pronation to prevent an incorrect range of motion measurement [27]. The Silfverskiöld test was performed to determine whether the equinus deformity was due to gastrocnemius tightness or another neuromuscular alteration. The test is considered positive if there is tension in the gastrocnemius when passive ankle dorsiflexion is limited or neutral with the knee extended during the application of moderate force under the forefoot. However, this loss of dorsiflexion normalizes when the knee is flexed, with a minimum difference of 13° [28, 29]. A lack of ankle dorsiflexion can predispose healthy patients to injuries such as genu recurvatum, early heel elevation, excessive pronation of the subtalar joint, metatarsalgia [30], ankle sprains, anterior tibial traction periostitis, medial tibial stress syndrome, Achilles tendinopathy, plantar fasciitis, anterior knee pain, gastrocnemius contracture, and anterior cruciate ligament injuries [31, 32, 33]. The range of motion of the talocrural joint was measured by the lead podiatrist researcher. Intraobserver and interobserver reliability of the range of motion was evaluated as in clinical practice, beginning from a 90° angle and assessing whether the range of motion was limited with both the knee extended and flexed, to differentiate between muscular tightness and bony impingement [34].

Subsequently, plantar pressures were taken by the lead podiatrist researcher using a portable digital pressure platform (Podoprint Aluminum V9.8 NL, TwinBox V8 44, Namrol, Barcelona, Spain). An automatic multistep calibration was performed before each measurement in accordance with the manufacturer's guidelines. The platform dimensions were 61.5 × 56.5 cm, with a thickness of 23 mm, weighing 3.15 kg, and containing 1600 resistive sensors (40 × 40). The sensitivity of the measurement was up to 1 Pa.

To determine the plantar pressure distribution, the software system divided the foot into three compartments: the first compartment corresponded to heel support, the second to midfoot support, and the third to forefoot support. An average of three readings was taken for each of the three compartments. To assess the plantar weight distribution pattern in both the healthy control group and the MS group, the average percentage plantar load for each compartment of the foot was calculated.

The equipment complied with the CE Declaration of Conformity and was calibrated a few days before the study began. The platform was connected via an interface unit to a personal computer running the PodoPrint data collection software, version 9.8 NL for Windows (TwinBox).

A study by Cobos‐Moreno et al. [35] demonstrated that the Podoprint pressure platform is a reliable tool for evaluating plantar pressure distribution in the biomechanical study of gait. Both the intraclass correlation coefficients (ICCs) and coefficient of variation (CVs) indicated excellent measurements across all areas of the foot evaluated. Besides, provided normative values that can serve as a reference for detecting pathological values; however, in our study, we also measured healthy patients, which allowed for comparison between their values and ours.

Static measurements were obtained for all participants in both groups, quantifying the following parameters: maximum plantar pressure (kPa) of each foot, percentage of body weight supported on each foot (%BW), and the area of the plantar surface (cm^2^) in contact with the platform.

For this study, the percentage of body weight (%BW) was calculated to assess plantar weight distribution. This was done by determining the percentage of plantar weight distribution for each compartment relative to the patient's body weight using the formula: weight [% C1 = (C1/Body weight) X 100] as described by Cavanagh et al. [36].

### 2.3 Statistical Analysis

We compared the demographic characteristics (age, weight, height, and BMI), foot pressure weights, and surfaces (PP, PBW, and PS) between the MS and healthy groups using a *t*‐test for two independent samples. Normality was assessed using the Kolmogorov–Smirnov test, and equality of variances was evaluated with Levene's test. If any of these prerequisites were not met, the nonparametric Mann–Whitney test was applied.

For all analyses, statistical significance was set at *p* < 0.05, with a 95% confidence interval. Data analysis was performed using the SPSS software, version 21.0 (SPSS Science, Chicago, Illinois).

## 3 Results

The total sample size was 88 participants, selected through convenience sampling, and divided into two groups: [1] patients with MS, serving as the case group (*n* = 44) and [2] participants without MS, forming the control group (*n* = 44). Participants were matched based on age, sex, foot size, and BMI, with cases and controls drawn from the same locality or healthcare area.

The characteristics of all participants, including both cases and controls, are presented in Table 1. This table displays the demographic variables—age, height, weight, and BMI. For these descriptive variables, no statistically significant differences were observed between the groups.

**TABLE 1:** Demographic characteristics and descriptive data of the participants in the multiple sclerosis (MS) group and the control group.

	Group	x̄ ± ơ	Sig. KS	Sig. Levene	Sig.
Age (years)	MS	47.3 ± 10.7	0.997	0.958	0.992^a^
	Healthy	47.3 ± 10.7	0.997		
Weight (kg)	MS	71.7 ± 13.8	0.572	0.385	0.484^a^
	Healthy	73.6 ± 12.5	0.985		
Height (cm)	MS	167.4 ± 8.9	0.364	0.652	0.875^a^
	Healthy	167.7 ± 8.7	0.177		
BMI (kg/m^2^)	MS	25.5 ± 4.0	0.363	0.458	0.401^a^
	Healthy	26.3 ± 4.6	0.295		

Abbreviations: BMI, body mass index; KS, Kolmogorov–Smirnov test.

^a^ Independent samples *t*‐test significance.

Table 2 presents the results of the measured variables for both the MS group and the healthy group. A significant decrease in peak pressure (PP) was observed in the MS group for both the right foot (*p* < 0.007) and the left foot (*p* < 0.001). However, no significant differences were found between the MS and healthy groups in the percentage of body weight supported (PBW) and plantar surface (PS) values for both the right and left foot (*p* > 0.05).

**TABLE 2:** Maximum peak pressure, percentage of body weight supported by each foot, and contact area of the plantar surface in the feet of the multiple sclerosis (MS) group and the control group.

		Group	x̄ ± ơ	Sig. KS	Sig. Levene	Sig.
PP (kPa)	Right foot	MS	65.1 ± 17.6	0.025^*^	0.631	0.007^b^ ^,^ ^*^
		Healthy	70.5 ± 13.2	0.742		
	Left foot	MS	66.7 ± 19.4	0.038^*^	0.303	0.001^b^ ^,^ ^*^
		Healthy	71.9 ± 10.5	0.947		
PBW (%)	Right foot	MS	51.6 ± 4.6	0.994	0.040^*^	0.407^b^
		Healthy	50.8 ± 3.3	0.159		
	Left foot	MS	48.5 ± 4.6	0.994	0.040^*^	0.407^b^
		Healthy	49.2 ± 3.3	0.159		
PS (cm^2^)	Right foot	MS	133.1 ± 30.7	0.508	0.113	0.078^a^
		Healthy	122.8 ± 22.7	0.870		
	Left foot	MS	125.6 ± 31.2	0.970	0.381	0.250^a^
		Healthy	118.7 ± 24.6	0.978		

Abbreviations: PP, peak pressure; PS, plantar surface; PWB, percentage of body weight.

^a^ Independent samples *t*‐test significant.

^b^ Mann–Whitney test significance.

^*^ Statistical significance.

The PS area in contact with the platform could potentially influence the distribution of loads on the foot and the maximum pressures recorded. Despite this, no statistically significant differences were detected between the MS and healthy groups in the PS area for the right foot (*p* = 0.078) or the left foot (*p* = 0.250).

Statistically significant differences were found in the percentage of body weight supported (PBW) between the MS and healthy groups for both feet (*p* = 0.407).

This study found that in patients with MS, the center of gravity is displaced toward the foot affected by the disease and is positioned more anteriorly. The centers of pressure for the right and left feet are not aligned; in the healthy foot, the center of pressure is anteriorly positioned relative to the center of gravity, whereas in the affected foot, the center of gravity is positioned more posteriorly. The point of maximum pressure (“M”) is consistently located in the rearfoot for both the affected and unaffected feet, typically skewed toward the right. The load distribution between the left and right foot shows a moderate shift toward the more affected side. In the healthy foot, 54% of the load is distributed to the forefoot compared to 50% in the forefoot of the affected foot. Additionally, the support surface area is larger in the affected foot, indicating a greater load‐bearing capacity.

## 4 Discussion

In this study, our hypothesis that patients with MS exhibit higher plantar pressures compared to healthy controls was not supported by the findings. Instead, this is the first study to identify significant differences in maximum static plantar pressure between patients with MS and healthy subjects, revealing that patients with MS experience lower peak pressures in both the right and left feet. This observation contradicts our initial hypothesis and suggests that MS leads to a redistribution of pressure that may result in an excess of ground reaction forces on certain foot structures.

By comparing patients with MS with healthy controls who were matched for age, sex, and BMI, we were able to minimize potential confounding factors, thereby isolating the impact of MS on plantar pressures. The results highlight the importance of focusing on the unique biomechanical challenges faced by patients with MS, particularly regarding the need to design and evaluate specialized footwear or insoles. Such interventions could help manage the altered pressure distribution, prevent foot complications, and ultimately improve the quality of life, independence, and overall welfare of patients with MS.

In our study, we did not observe a noticeable shift in load toward the unaffected foot, which could result in uneven loading between the left and right feet during static stance. This uneven distribution was particularly evident in the maximum pressure observed in the forefoot. This finding is inconsistent with previous studies that have reported asymmetry in pressure distribution between the feet in patients with MS. Such asymmetry may indicate compensatory mechanisms developed by patients to manage their condition as well as the impact of MS on postural stability and balance. Understanding these patterns is crucial for designing targeted interventions, such as custom footwear or orthotics, aimed at reducing the risk of further complications and enhancing overall mobility and quality of life for patients with MS [37].

Several studies have emphasized the utility of pressure platforms in detecting early signs of ataxia and using this tool in clinical practice for early diagnosis [38, 39]. Baropodometry, in particular, has proven effective in identifying key biomechanical issues. For instance, increased pressures, supported body weight, and contact area in the affected foot can serve as early indicators of drop foot in patients with MS. This tool allows clinicians to monitor and intervene early, potentially mitigating further complications [3].

Another crucial aspect in patients with MS is the stability and compensatory mechanisms employed to maintain balance during static posture. In healthy patients, the center of gravity typically remains centered and rarely shifts anteriorly. However, in patients with MS, the center of gravity is often displaced anteriorly and shifted toward the unaffected lower limb. This shift likely represents a compensatory mechanism to reduce the load and pressure on the foot that first experienced the onset of MS.

These findings differ with those of Neamtu et al. [40] who could be discrepancies between the right and left sides and the development of compensatory strategies to maintain balance and stability in patients with MS.

The study has several limitations that must be considered when interpreting the results. Firstly, the limited availability of subjects with MS in the study area may have impacted the representativeness of the sample and, consequently, the generalizability of the findings to a broader MS population. This limitation could restrict the applicability of the results to different demographics or geographic regions. Secondly, the exclusion of participants who could not maintain a bipedal stance without assistance may have introduced bias, omitting subpopulations with relevant characteristics, such as those with more severe mobility impairments. Thirdly, the study did not account for the value of interactions between fixed factors, such as the relationship between foot dominance and plantar pressure distribution, nor did it statistically analyze changes in center of gravity alignment. These omissions could limit our understanding of how these variables interact and influence the outcomes observed in patients with MS.

These limitations highlight important areas for future research. Addressing these methodological challenges in subsequent studies could enhance the validity and reliability of findings, offering a more comprehensive understanding of plantar pressure distribution, balance, and gait in patients with MS. This could ultimately lead to more effective clinical interventions and improved quality of life for patients.

## 5 Conclusion

In this study, it was observed that reduction in maximum peak pressures and weight‐bearing capacity in patients with MS suggests diminished plantar pressure, which may be indicative of altered gait patterns and balance issues. These findings emphasize the importance of developing targeted interventions to correct these imbalances and improve mobility and stability for individuals with MS.

## Author Contributions

**Francisco Javier Ruiz‐Sánchez:** conceptualization, data curation, formal analysis, investigation, methodology, resources, visualization, writing – review and editing. **Maria do Rosário Martins:** conceptualization, data curation, formal analysis, methodology, resources, writing – review and editing. **Marta Elena Losa‐Iglesias:** conceptualization, formal analysis, methodology, resources, writing – review and editing. **Ricardo Becerro‐de‐Bengoa‐Vallejo:** conceptualization, formal analysis, methodology, writing – review and editing. **Juan Gómez‐Salgado:** conceptualization, formal analysis, methodology, resources, writing – review and editing. **Miguel Ángel Saavedra‐García:** conceptualization, formal analysis, investigation, methodology, resources, visualization, writing – review and editing. **Daniel López‐López:** conceptualization, formal analysis, investigation, methodology, resources, visualization, writing – review and editing. **Ana María Jiménez‐Cebrián:** conceptualization, formal analysis, methodology, resources, visualization, writing – review and editing.

## Ethics Statement

The study adhered to the principles outlined in the Declaration of Helsinki and received approval from the Ethics Committee of the Provincial Research of Malaga with register number CEUMA: 32‐2021‐H.

## Consent

All subjects involved in the study provided written informed consent.

## Conflicts of Interest

The authors declare no conflicts of interest.

## Data Availability

The dataset underpinning the findings of this article can be requested by contacting f.ruiz@udc.es at the Research, Health, and Podiatry Group, Department of Health Sciences, Faculty of Nursing and Podiatry, Industrial Campus of Ferrol, Universidade da Coruña, 15403 Ferrol, Spain.
